# Establishing Decisional Cutoff Values of Neurofilament Light Chains in Cerebrospinal Fluid Measured by Fully Automated Chemiluminescent Enzyme Immunoassay

**DOI:** 10.1002/jcla.25152

**Published:** 2025-01-15

**Authors:** Luisa Agnello, Caterina Maria Gambino, Fabio Del Ben, Anna Maria Ciaccio, Concetta Scazzone, Marcello Ciaccio

**Affiliations:** ^1^ Department of Biomedicine, Neurosciences and Advanced Diagnostics, Institute of Clinical Biochemistry, Clinical Molecular Medicine, and Clinical Laboratory Medicine University of Palermo Palermo Italy; ^2^ Department of Laboratory Medicine University Hospital Paolo Giaccone Palermo Italy; ^3^ Immunopathology and Cancer Biomarkers Centro di Riferimento Oncologico (CRO)‐IRCCS Aviano Italy; ^4^ Internal Medicine and Medical Specialties “G. D'Alessandro", Department of Health Promotion, Maternal and Infant Care University of Palermo Palermo Italy

**Keywords:** Alzheimer's disease, CSF, Fujirebio, Lumioulse, Neurofilament

## Abstract

**Introduction:**

Neurofilament light chain (NfL) is one of the most important biomarkers in the field of clinical neurochemistry. Several analytical methods have been developed in the last decade. Recently, Fujirebio introduced a ready‐to‐use assay kit for measuring NfL levels in the cerebrospinal fluid (CSF) on the fully automated LUMIPULSE G System. In this study, we established the decisional cutoffs for CSF NfL.

**Materials and Methods:**

We performed a retrospective observational study including patients with cognitive decline. CSF NfL levels were measured by two analytical methods: the NF‐light ELISA kit (UmanDiagnostics) and the Lumipulse G1200 fully automated system (Fujirebio). We calculated the cutoffs for the Lumipulse, starting from the consolidated cutoffs of the ELISA method for each age and using the equation obtained by the regression analysis.

**Results:**

The study population consisted of 100 patients with cognitive decline. The median levels of CSF NfL measured by Lumipulse and ELISA were 776.5 ± 772.6 pg/mL and 473.5 ± 443.5 pg/mL, respectively, significantly different (*p* < 0.001). The Spearman's rank correlation coefficient was 0.962, indicating a robust positive correlation between the two measurement methods. The equation derived from the Passing–Bablok regression analysis was CSF CLEIA = −61.16 + 1.83 × CSF ELISA. Based on this equation, we defined the decisional cutoff values.

**Conclusions:**

Decisional cutoffs are fundamental tools for guiding clinicians to use biomarkers' results and interpretation appropriately. This is the first study establishing the decisional cutoff value of NfL measured by Lumipulse, a fully automated platform widely used in clinical laboratories.

## Graphical abstract

The immunoassays methods to measure neurofilament light chain in cerebrospinal fluid has undergone an important evolution. Establishing decisional cut‐off values is mandatory for introducing a biomarker in clinical practice.

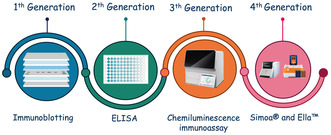

## 1 Introduction

Neurofilament light chain (NfL) is a subunit of the neurofilaments, which are intermediate filaments located in myelinated axons of the central and peripheral nervous systems (CNS and PNS). Neurofilaments consist of four different subunits: neurofilament heavy chain (NfH), medium chain (NfM), NfL and alpha‐internexin in the CNS or peripherin in the PNS [1]. Among these, NfL is the most abundant and soluble.

**FIGURE 1:**
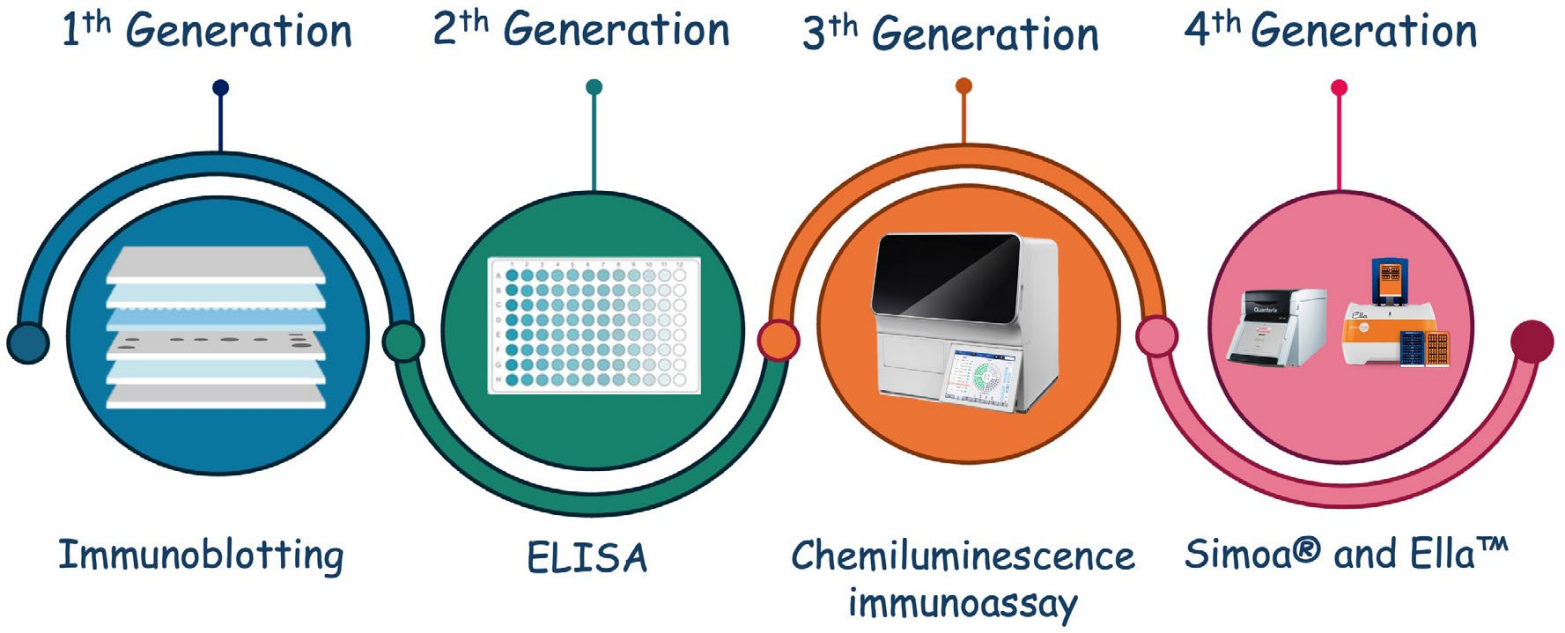
Evolution of immunoassay methods to measure neurofilament light chain in cerebrospinal fluid.

NfL is regarded as a highly sensitive but not specific biomarker of neuroaxonal injury. An increase in its levels indicates neuronal damage but not the underlying etiology. Thus, in clinical practice, it could be helpful to (i) identify early neurodegenerative processes, (ii) quantitatively assess the degree of active brain pathology [2], (iii) predict clinical outcomes and (iv) monitor treatment. Additionally, in clinical trials, it is commonly deployed as an outcome measure in different diseases, including Alzheimer's disease (AD), multiple sclerosis and amyotrophic lateral sclerosis [3]. The quantification of NfL levels relies on two types of analytical methods: immunoassay and mass spectrometry. Immunoassays are the most widely used worldwide and over time have undergone a significant technological evolution (Figure 1). Through different technologies, immunoassays rely on the ability of antibodies to recognize targets by binding to specific epitopes in complex biological solutions [4].

In March 2023, Fujirebio launched the ready‐to‐use assay kit for measuring CSF NfL on the fully automated LUMIPULSE G System. Lumipulse is one of the most widely used automatic platforms for measuring AD core biomarkers across the world [5]. Currently, no decisional cutoff value is available. However, establishing decisional cutoff values is mandatory when introducing a biomarker into clinical practice. This is challenging for CSF biomarkers due to the CSF collection's invasiveness. Indeed, for some CSF biomarkers, such as AD biomarkers, to overcome this important limitation, Authors included patients with other neurodegenerative or neurological diseases as controls. However, this cannot be applied to NfL because their levels increase in different neurological diseases, including neurodegenerative and neuroinflammatory diseases, psychiatric disorders and non‐primary neurological diseases, such as COVID‐19 and hypoxic brain injury [2, 3]. Indeed, NfL has been defined as the “neurologist's troponin” and “the c‐reactive protein of neurology” [6, 7]. To overcome these critical limitations, in this study, we developed a mathematical formula to define the decisional cutoff value of CSF NfL measured by Lumipulse, starting from the decisional cutoff value established by UmanDiagnostic enzELISA.

## 2 Materials and Methods

### 2.1 Study Population

We performed a retrospective observational study at the University Hospital “P. Giaccone”, Palermo, Italy, including patients with cognitive decline who underwent lumbar punctures for CSF collection as a part of the routine clinical diagnostic workup.

All samples were analyzed at the Institute of Clinical Biochemistry, Clinical Molecular Medicine, and Clinical Laboratory Medicine, Department of Biomedicine, Neurosciences and Advanced Diagnostics, University of Palermo, Palermo, Italy.

According to recent guidelines, an expert neurologist made the diagnosis of AD or other neurological diseases based on medical history, clinical examination, neuropsychological testing, neuroimaging, fluorodeoxyglucose positron emission tomography (PET) and CSF biomarker findings [8, 9, 10].

All clinical and biological assessments were carried out in accordance with the Declaration of Helsinki, and the local ethics committee approved the study (Nr. 02/2023). All participants gave written consent. The informed consent states that “the biological material (collected) may also be used for research purposes.”

### 2.2 Laboratory Analysis

We measured NfL levels in the CSF of all patients. CSF was obtained by a lumbar puncture at the L3/4 or L4/5 interspace using a 21‐gauge needle. It was collected in polypropylene tubes, centrifuged at 500 *g* for 20 min, aliquoted in polypropylene tubes and stored at −80°C until analysis, according to international consensus protocols [11].

CSF NfL levels were measured using two analytical methods: NF‐light ELISA kit (UmanDiagnostics, Umeå, Sweden; catalog number: 10–7002) and CLEIA on the Lumipulse G1200 fully automated system (Fujirebio Inc., Tokyo, Japan).

The NF‐light ELISA kit was employed according to the manufacturer's instructions. Calibrators and samples were run in duplicate, and the mean of the duplicate values was used as the final readout. All samples were diluted twofold and run without information on any clinical data.

The standard curve of the ELISA assay covers the interval of 50–5000 pg/mL. The kit manufacturers stated that the limits of detection (LoD) and quantification (LoQ) are 33 and 81 pg/mL, respectively. The intra‐assay coefficient of variation is < 5%, and the inter‐assay coefficient of variation is < 10%.

CSF NfL levels were analyzed on the Lumipulse G1200 using the Lumipulse G NfL CSF kit. The kit manufacturer stated that the LoD and LoQ for CSF NfL are 4 and 6 pg/mL, respectively. The intra‐assay coefficient of variation is < 7%. Calibrators and samples were run in duplicates, and the mean of the duplicate values was used as the final readout. No dilution of the sample was required.

### 2.3 Statistical Analysis

The data distribution was visually inspected using histograms and Q‐Q (quantile‐quantile) plots for both CSF CLEIA and CSF ELISA measurements. In addition to visual inspection, the Shapiro–Wilk test was employed to evaluate the normality of the data. The results from these tests indicated that the data did not strictly follow a normal distribution. The CSF CLEIA and CSF ELISA measurement methods were compared using the Passing–Bablok regression analysis. This nonparametric regression method is particularly suited for method comparison studies as it does not assume a specific distribution for the data and is robust to outliers. Spearman correlation coefficient was calculated as well. Descriptive statistics are presented in the form of median and interquartile ranges. The significance level was set to *p* < 0.05.

## 3 Results

The study population consisted of 100 patients with cognitive decline: 41 patients with AD and 59 patients with other types of cognitive decline, including frontotemporal dementia, Creutzfeldt‐Jakob disease and vascular dementia. 46 patients were male and 54 female, with an average age of 70 ± 9.2 years.

The median levels of CSF NfL measured by CLEIA and ELISA were 776.5 ± 772.6 pg/mL and 473.5 ± 443.5 pg/mL, respectively, significantly different (*p* < 0.001).

The Spearman's rank correlation coefficient (*ρ*) was 0.962, indicating a very strong positive correlation between the two measurement methods.

**FIGURE 2:**
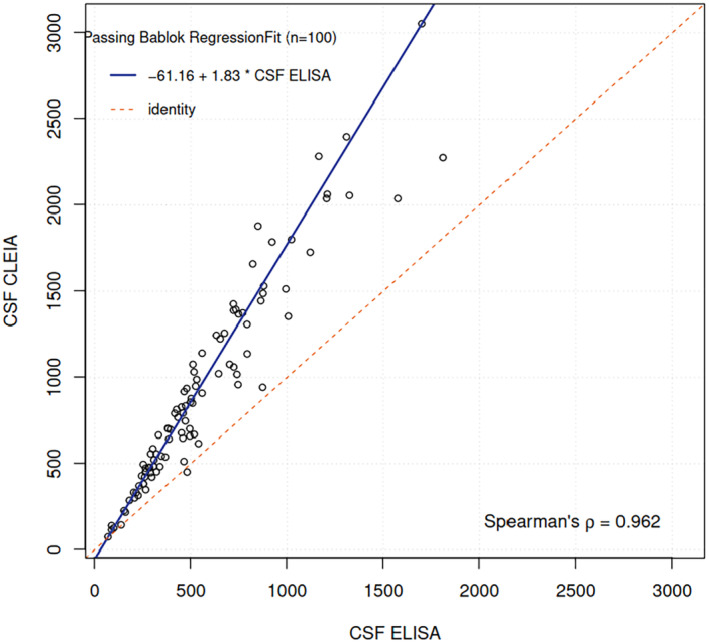
Passing–Bablok regression analysis of the correlation between ELISA and CLEIA for measuring NfL in the CSF. The regression line is shown in blue on the scatter plot, and the identity line (where CSF CLEIA equals CSF ELISA) is indicated by the red dashed line. Right: The residual plot against the mean of the observed values from both methods (CSF CLEIA and CSF ELISA).

The relationship between CSF CLEIA and CSF ELISA measurements was assessed using Passing–Bablok regression analysis in 100 data points (Figure 2). The regression equation derived was CSF CLEIA = −61.16 + 1.83 × CSF ELISA.

The residuals appear relatively consistent across the range of measurements and are equally spread around zero, suggesting that the relationship between CSF CLEIA and CSF ELISA is linear, stable and well‐described by the Passing–Bablok regression.

These findings demonstrate a significant and robust linear relationship between the CLEIA and ELISA assays, with a consistent positive bias of CLEIA compared to ELISA. In other words, CLEIA values tend to be higher than ELISA values.

Based on this, we calculated the cutoffs for the CLEIA method, starting from the consolidated cutoffs of the ELISA method for each age and using the equation provided by the regression analysis. Results are displayed in Table 1.

**TABLE 1:** Decisional cutoff value of CSF NfL measured by ELISA and CLEIA.

Age, years	NfL, pg/mL—ELISA	NfL, pg/mL—CLEIA
< 30	> 380	> 638
30–39	> 560	> 971
40–59	> 890	> 1581
≥ 60	> 1850	> 3357

## 4 Discussion

Identifying neuroaxonal injury and quantifying its severity is crucial in the patient care path, as it aids in diagnosing and, especially, the prognosis of neurological conditions. NfL is a tissue‐ but not disease‐specific biomarker that provides objective and quantitative measures of neuroaxonal loss. Increased CSF NfL levels were first reported in neurodegenerative conditions over 20 years ago [12]. Nowadays, it is regarded as one of the most important biomarkers in clinical neurochemistry [13]. Immunoblotting techniques were the first immunoassay allowing the semiquantitative measurement of NfL in CSF [14]. The ELISA developed by Rosengren et al. [12] in 1996 provided the first quantitative measurement of CSF NfL. Then, several ELISA methods have been developed, including the commercially available UmanDiagnostics assay. Successively, more sensitive third‐generation methods, including particle‐based chemiluminescence immunoassay technology, such as CLEIA and electrochemiluminescence immunoassay (ECLIA), have been commercialized, making possible the detection of NfL also in blood. Finally, third‐generation ultrasensitive immunoassay, including Simoa (Quanterix) and Ella (Bio‐Techne), allows measuring with high accuracy NfL in different biological fluids [15, 16]. Simoa is a digital immunoassay using two highly specific, noncompeting monoclonal antibodies to detect single molecules attached to paramagnetic beads, achieving sensitivity down to femtomolar concentrations. Ella is a microfluidic cartridge‐based immunoassay platform measuring up to 72 samples in triplicate inside glass nanoreactors using a fluorescent substrate. Each analytical method has advantages and disadvantages. Although fourth‐generation methods are ultrasensitive, they are costly and require dedicated instrumentation and specialized personnel. On the other hand, second‐ and third‐generation methods are less sensitive but are affordable and easier to use allowing their widespread diffusion in clinical practice. Several authors tested the correlation of NfL levels measured by the different analytical methods, showing significant differences, highlighting the need for assay‐specific decisional cutoffs to differentiate between pathological and non‐pathological conditions [17, 18]. Decisional cutoffs are fundamental tools for guiding clinicians to use biomarkers' results and interpretation appropriately. The Institute of Medicine defines a biomarker cutoff as a “specified quantitative measure used to demarcate the presence or absence of a health‐related condition; often used in interpreting measures obtained from blood analysis” [19]. Various statistical methods for optimal cut‐point selection have been proposed, with the receiver operator characteristic (ROC) curve analysis and relative tools, such as the Youden index, being the method of choice [20]. The calculation of ROC curve analysis requires data from two independent groups, healthy and diseased individuals. In the field of clinical neurochemistry, and especially for CSF biomarkers, the biological matrix collection from healthy individuals is challenging. Thus, in this study, we used an alternative approach to establish decisional cutoffs of CSF NfL measured by the fully automated platform Lumipulse. Specifically, we applied a mathematical formula starting from the decisional cutoff of CSF NfL measured by the ELISA UmanDiagnostics. This choice relies on the following assumptions: (i) UmanDiagnostic ELISA and Lumipulse Kit assays use the same antibodies produced by UmanDiagnostics to detect the biomarker [21, 22]; (ii) a very robust correlation of UmanDiagnostic ELISA and Lumipulse for measuring NfL in CSF has been found; and (iii) UmanDiagnostic ELISA is the only approved for use in in vitro diagnostics.

The findings of this study are of significant clinical relevance for several reasons. First, decisional cutoffs of NfL have a critical role in clinical practice by helping clinicians make informed, standardized and evidence‐based decisions regarding diagnosis, treatment and management across a wide range of neurological diseases. Overall, the establishment of decisional cutoffs enables the practical use of a biomarker in clinical settings. Given the substantial growth of NfL in recent years and the leadership of Fujirebio in neurochemistry biomarkers, the provision of decisional cutoffs will facilitate the integration of NfL into routine labs. The fully automated random‐access LUMIPULSE G immunoassay system, with its advantages over other immunoassay‐based systems, such as rapid and straightforward measurement (within 35 min), further supports this implementation. Second, our analysis underscores that while the correlation between methods is robust, the decisional cutoff cannot be used interchangeably. This is due to the different detection methods, each offering a unique analytical sensitivity.

The next crucial step is to validate the established decisional cutoffs in real‐world clinical practice. This ongoing work is of paramount importance, as it will further confirm the utility and reliability of these cutoffs in guiding the use and interpretation of biomarker results in clinical settings.

## Ethics Statement

The study was approved by the local ethics committee (Nr. 02/2023).

## Consent

All patients gave written consent.

## Conflicts of Interest

The authors declare no conflicts of interest.

## Data Availability

The data that support the findings of this study are available from the corresponding author, upon reasonable request.

## References

[1] A. Yuan , M. V. Rao , and N. R. A. Veeranna , “Neurofilaments and Neurofilament Proteins in Health and Disease,” Cold Spring Harbor Perspectives in Biology 9 (2017): a018309. 28373358 10.1101/cshperspect.a018309 PMC5378049

[2] F. Bavato , C. Barro , L. K. Schnider , et al., “Introducing Neurofilament Light Chain Measure in Psychiatry: Current Evidence, Opportunities, and Pitfalls,” Molecular Psychiatry 29 (2024): 2543–2559, 10.1038/s41380-024-02524-6. 38503931 PMC11412913

[3] C. A. Leckey , J. B. Coulton , T. A. Giovannucci , et al., “CSF Neurofilament Light Chain Profiling and Quantitation in Neurological Diseases,” Brain Communications 6 (2024): fcae132. 38707707 10.1093/braincomms/fcae132 PMC11069115

[4] S. Hörber , P. Achenbach , E. Schleicher , and A. Peter , “Harmonization of Immunoassays for Biomarkers in Diabetes Mellitus,” Biotechnology Advances 39 (2020): 107359. 30802485 10.1016/j.biotechadv.2019.02.015

[5] A. Silva‐Spínola , M. J. Leitão , A. Nadal , N. Le Bastard , I. Santana , and I. Baldeiras , “Exploring the Potential of Fully Automated LUMIPULSE G Plasma Assays for Detecting Alzheimer's Disease Pathology,” Alzheimer's Research & Therapy 16 (2024): 51. 10.1186/s13195-024-01397-9 PMC10918996 38454502

[6] S. Thebault , R. A. Booth , and M. S. Freedman , “Blood Neurofilament Light Chain: The Neurologist's Troponin?,” Biomedicine 8 (2020): 523. 10.3390/biomedicines8110523 PMC7700209 33233404

[7] K. L. Lambertsen , C. B. Soares , D. Gaist , and H. H. Nielsen , “Neurofilaments: The C‐Reactive Protein of Neurology,” Brain Sciences 10 (2020): 56. 31963750 10.3390/brainsci10010056 PMC7016784

[8] G. M. McKhann , D. S. Knopman , H. Chertkow , et al., “The Diagnosis of Dementia Due to Alzheimer's Disease: Recommendations From the National Institute on Aging‐Alzheimer's Association Workgroups on Diagnostic Guidelines for Alzheimer's Disease,” Alzheimer's & Dementia 7, no. 3 (2011): 263–269. 10.1016/j.jalz.2011.03.005 PMC3312024 21514250

[9] M. S. Albert , S. T. DeKosky , D. Dickson , et al., “The Diagnosis of Mild Cognitive Impairment Due to Alzheimer's Disease: Recommendations From the National Institute on Aging‐Alzheimer's Association Workgroups on Diagnostic Guidelines for Alzheimer's Disease,” Alzheimer's & Dementia 7, no. 3 (2011): 270–279. 10.1016/j.jalz.2011.03.008 PMC3312027 21514249

[10] C. R. Jack, Jr. , D. A. Bennett , K. Blennow , et al., “NIA‐AA Research Framework: Toward a Biological Definition of Alzheimer's Disease,” Alzheimer's & Dementia 14, no. 4 (2018): 535–562. 10.1016/j.jalz.2018.02.018 PMC5958625 29653606

[11] O. Hansson , “Biomarkers for Neurodegenerative Diseases,” Nature Medicine 27 (2021): 954–963. 10.1038/s41591-021-01382-x 34083813

[12] L. E. Rosengren , J. E. Karlsson , J. O. Karlsson , L. I. Persson , and C. Wikkelsø , “Patients With Amyotrophic Lateral Sclerosis and Other Neurodegenerative Diseases Have Increased Levels of Neurofilament Protein in CSF,” Journal of Neurochemistry 67 (1996): 2013–2018. 8863508 10.1046/j.1471-4159.1996.67052013.x

[13] M. Khalil , C. E. Teunissen , S. Lehmann , et al., “Neurofilaments as Biomarkers in Neurological Disorders—Towards Clinical Application,” Nature Reviews. Neurology 20, no. 5 (2024): 269–287. 38609644 10.1038/s41582-024-00955-x

[14] S. Coppens , S. Lehmann , C. Hopley , and C. Hirtz , “Neurofilament‐Light, a Promising Biomarker: Analytical, Metrological and Clinical Challenges,” International Journal of Molecular Sciences 24 (2023): 11624. 37511382 10.3390/ijms241411624 PMC10380627

[15] R. Hendricks , D. Baker , J. Brumm , et al., “Establishment of Neurofilament Light Chain Simoa Assay in Cerebrospinal Fluid and Blood,” Bioanalysis 11, no. 15 (2019): 1405–1418. 31401845 10.4155/bio-2019-0163

[16] M. Truffi , M. Garofalo , A. Ricciardi , et al., “Neurofilament‐Light Chain Quantification by Simoa and Ella in Plasma From Patients With Dementia: A Comparative Study,” Scientific Reports 13 (2023): 4041. 36899015 10.1038/s41598-023-29704-8 PMC10006166

[17] L. M. Yee , T. G. Lively , and L. M. McShane , “Biomarkers in Early‐Phase Trials: Fundamental Issues,” Bioanalysis 10 (2018): 933–944. 29923753 10.4155/bio-2018-0006 PMC6123886

[18] C. Ferreira‐Atuesta , S. Reyes , G. Giovanonni , and S. Gnanapavan , “The Evolution of Neurofilament Light Chain in Multiple Sclerosis,” Frontiers in Neuroscience 15 (2021): 642384. 33889068 10.3389/fnins.2021.642384 PMC8055958

[19] Institute of Medicine , Dietary Reference Intakes for Calcium and Vitamin D (Washington: National Academies Press, 2010). 21796828

[20] M. Hassanzad and K. Hajian‐Tilaki , “Methods of Determining Optimal Cut‐Point of Diagnostic Biomarkers With Application of Clinical Data in ROC Analysis: An Update Review,” BMC Medical Research Methodology 24 (2024): 84. 38589814 10.1186/s12874-024-02198-2 PMC11000303

[21] S. Das , N. Dewit , D. Jacobs , et al., “A Novel Neurofilament Light Chain ELISA Validated in Patients With Alzheimer's Disease, Frontotemporal Dementia, and Subjective Cognitive Decline, and the Evaluation of Candidate Proteins for Immunoassay Calibration,” International Journal of Molecular Sciences 23 (2022): 7221. 35806226 10.3390/ijms23137221 PMC9266977

[22] B. Arslan and H. Zetterberg , “Neurofilament Light Chain as Neuronal Injury Marker—What Is Needed to Facilitate Implementation in Clinical Laboratory Practice?,” Clinical Chemistry and Laboratory Medicine 61 (2023): 1140–1149. 36880940 10.1515/cclm-2023-0036

